# A Genome-Wide Association Study Reveals QTLs and Candidate Genes Associated with the Carotenoid Content in the Flesh of *Cucurbita pepo* L. Fruit

**DOI:** 10.3390/antiox14091090

**Published:** 2025-09-05

**Authors:** Alba López, Alicia García, Alejandro Castro-Cegrí, María Segura, Álvaro Benítez, Francisco Palma, Dolores Garrido, Cecilia Martínez, Manuel Jamilena

**Affiliations:** 1 Department of Biology and Geology, Agri-Food Campus of International Excellence (CeiA3) and Research Center CIAIMBITAL, University of Almería, 04120 Almería, Spain; alf893@ual.es (A.L.);; 2 Department of Plant Physiology, University of Granada, 18071 Granada, Spain; 3 Department of Agroforestry Sciences, ETSI, University of Huelva, 21007 Huelva, Spain

**Keywords:** squash, genetic control, carotenoid pathway, genomic prediction, natural variation, nutraceutical quality, germplasm

## Abstract

Considering the importance of carotenoids in the human diet, their enhancement is a key trait in current breeding programs. This study assessed lutein, zeaxanthin, α-carotene, and β-carotene levels in the flesh of mature fruits from 257 global *C. pepo* accessions. Lutein and β-carotene were the most prevalent, with top accessions identified for each carotenoid. A panel of 120 accessions with reliable carotenoid contents and genetic diversity was analyzed using 23,111 GBS-generated SNPs in genome-wide association studies (GWAS). Three genomic regions (*qtl1, qtl3,* and *qtl13*) on chromosomes 1, 3, and 13 were significantly linked to carotenoid levels, with alternative alleles increasing the carotenoid content, leading to yellowish–orange flesh. Seven candidate genes were identified: *CpTIC56*, *CpHSHP70*, and *CpPDL8*, which regulate carotenoid biosynthesis in chloroplasts; *CpSPX* and *CpPHO1*, associated with phosphate homeostasis and carotenoid buildup; *CpMYB106*, co-expressed with carotenoid biosynthesis genes; and a *CpPPR* RNA-binding protein. RNA-seq data from yellow- and white-fleshed fruits supported their involvement in carotenoid accumulation. These results improve our understanding of the genetic control of carotenoid buildup in *C. pepo* fruit, supporting breeding efforts for improved nutritional quality.

## 1. Introduction

Carotenoids are essential isoprenoid compounds (C40H56) that are synthesized by all photosynthetic organisms, including plants, algae, certain fungi, and bacteria [1]. They are classified into two main groups: carotenes (hydrocarbons) and xanthophylls (oxygenated). The best-known carotenes are α-carotene, β-carotene, and lycopene, while lutein, zeaxanthin, and violaxanthin are examples of xanthophylls [2]. In plants, carotenoids and their derivatives are essential for photosynthesis, photoprotection against oxidation, pigmentation (yellow, orange, and red tissue coloring), signaling, and phytohormone synthesis (precursors for the production of the plant hormones abscisic acid and strigolactones) [3,4,5]. In addition, carotenoids have multiple functions as antioxidants, help attract pollinators, and ensure pollen grain transfer [6].

Most animals lack the biosynthetic mechanism required to produce carotenoids; therefore, they must obtain these natural compounds from fruits and vegetables [7]. In humans, dietary carotenoids are thought to act as antioxidants and anti-inflammatories and are involved in many aspects of human health, such as protection against cardiovascular disease, cancer, diabetes, cataracts, aging, ocular disease, skin damage from UV radiation and other diseases, neurodegenerative diseases, and age-related macular degeneration [7,8]. Commercially, carotenoids are also used in the production of nutraceutical foods, cosmetics, and dietary supplements due to their health-promoting properties [9,10]. β-carotene is of particular relevance as it is a precursor to vitamin A, which is essential for vision, the immune system, and cell growth. Vitamin A deficiency affects millions of people worldwide, particularly children in developing countries.

Pumpkins and squashes (genus *Cucurbita*) are members of the Cucurbitaceae family. They are native to Latin America but have been cultivated in Europe for more than 500 years. The genus *Cucurbita* comprises five domesticated species (*Cucurbita argyrosperma* (Huber), *Cucurbita ficifolia* (Bouche), *Cucurbita maxima* (Duchesne), *Cucurbita moschata* (Duchesne), and *Cucurbita pepo* (L.), of which *Cucurbita moschata*, *Cucurbita maxima*, and *Cucurbita pepo* (L.) are the most economically important cultivated species worldwide [11]. The species *Cucurbita pepo* (L.) is highly polymorphic in both plant structure and fruit shape, size, and rind and flesh color. The flesh exhibits a wide range of colors, including white, cream, yellow, and orange [12]. The predominant carotenoids in the fruits are α- and β-carotene, β-cryptoxanthin, lutein, and zeaxanthin [13,14]. The color of the flesh of the fruit is positively correlated with carotenoid content, and a particular hue of the flesh can be related to the proportion of individual carotenoids [15]. It is therefore evident that carotenoids are regarded as secondary metabolites within the chromoplasts of the fruits and flowers, playing a pivotal role in imparting the diverse colors observed in these tissues [16]. The composition of carotenoids in a given organ is dependent on a number of factors, including the cultivar, growing conditions, harvest time, and storage conditions [17,18,19].

The biosynthesis of carotenoids in cucurbits occurs in differentiated plastids, primarily in the chloroplasts and chromoplasts of flower petals, fruits, and roots [20,21]. Carotenoids can be considered secondary metabolites in chromoplasts, yet they are essential for photosynthesis within chloroplasts [16]. This intricate metabolic pathway entails a cascade of enzymes that facilitate the conversion of isoprenoid precursors into the ultimate carotenoids. The process begins with the formation of geranylgeranyl pyrophosphate (GGPP), from which lycopene, the precursor to all other carotenoids, is synthesized [22]. Lycopene can be converted to β-carotene, α-carotene, and other carotenes, or to xanthophylls such as lutein, zeaxanthin, and violaxanthin [23].

Currently, genotyping by sequencing (GBS) technology is arguably the most widely used method for genetically characterizing plant germplasm [24]. Single-nucleotide polymorphisms (SNPs) identified by GBS are employed in genome-wide association studies (GWAS) to ascertain allelic variants in genes that regulate significant agronomic and nutritional traits in plants [25]. GWAS, based on linkage disequilibrium (LD), represent an effective complement strategy to quantitative trait locus (QTL) mapping in the dissection of associations between genotypes and phenotypes in germplasm collections [26,27,28].

While carotenoid content is a prioritized trait in current commercial breeding programs, the variation in this trait among accessions and its underlying genetic control remain uncertain. The present study evaluated the levels of lutein, zeaxanthin, α-carotene, and β-carotene, as well as the flesh color, in mature fruits from 257 *C. pepo* accessions sourced from around the world. A subset of 120 accessions, selected on the basis of their reliable carotenoid content and genetic diversity, was then utilized in genome-wide association studies (GWAS). A total of 23,111 GBS-generated SNPs in the aforementioned diversity panel were employed in GWAS, identifying potential genomic regions, molecular markers and genes associated with these traits in the crop and providing a valuable genomic resource for global programs.

## 2. Materials and Methods

### 2.1. Germplasm and Genomic Data Used for GWAS

A collection of 257 accessions was evaluated for the contents of α-carotene, β-carotene, zeaxanthin, and lutein. The accessions were selected from a collection of over 800 accessions provided by the National Plant Germplasm System of the United States Department of Agriculture (USDA, Washington, DC, USA) to encompass the full range of geographical regions and mature flesh colors observed in squash. Genomic data were obtained from CuGenDB (http://cucurbitgenomics.org/), which hosts SNP data from 830 accessions generated by genotyping by sequencing (GBS). Additional information regarding each accession was obtained from the USDA Germplasm Resources Information Network (GRIN) database (www.ars-grin.gov) and is provided in Table S1.

The plants of each accession were grown in a multispan greenhouse during the spring–summer of 2022, situated in the Center for Innovation and Technology “Fundación UAL-ANECCOP” in Almería, Spain (latitude: 36°51′53.2″ N, longitude: 2°16′58.8″ W, altitude: 87 m). The plants were cultivated in “enarenado,” a stratified sand-mulched soil system commonly used in Almería’s greenhouse agriculture [29]. The greenhouse was passively ventilated with flap roof windows and lateral side panels. A combined drip irrigation and fertigation system was used. According to Sonneveld and Straver [30], a standard nutrient solution was used for EC 2.2 (dS/m) based on the ionic nutrient balance. The pH was adjusted to 5.8 by adding diluted nitric acid. Fertigation was uniformly applied via drip irrigation with drip tape arranged in paired lines. There was 0.5 m spacing between lines within each pair and 1.5 m spacing between adjacent pairs of lines. Within drip lines, there was 0.5 m spacing between drip emitters, providing an emitter density of two emitters per m^2^. Each emitter discharged 3 L per hour. Individual plants were positioned directly next to each emitter, resulting in a spacing of two plants per m^2^. All cultural practices were conducted according to local crop management practices, which were in turn based on the best available scientific knowledge.

### 2.2. Carotenoid Extraction and Quantification

The carotenoid content was quantified in flesh-mature fruits at 50 days after pollination using a minimum of three biological replicates per accession. Those accessions whose carotenoid content was found to be very variable between fruits of the same accession, and those from which an insufficient number of fruits were obtained, were excluded from the study. The extraction of carotenoids was conducted according to the methodology proposed by Wald et al. [31], with certain modifications. A total of 30 mg of lyophilized tissue was extracted using 0.6 mL of hexane containing butylated hydroxytoluene (1 mM) and trans-β-apo-8′-carotenal as an internal standard. Samples were saponified with 1 M potassium hydroxide solution for a period of 3 h. Subsequently, a saturated sodium chloride solution was added. The extract was vortexed for 15 s and centrifuged at 9000× *g* for 5 min. The nonpolar phase was then removed and dried under a nitrogen flow. Finally, the residue was redissolved in isopropanol.

The analysis of α-carotene, β-carotene, and lutein was conducted using reversed-phase high-performance liquid chromatography–photodiode array (RP-HPLC-PDA) using an Agilent 1260 Infinity system equipped with a C30 YMC-carotenoid column (3 μm, 250 × 4.6 mm), in accordance with the methodology outlined by Castro-Cegrí et al. [32]. The mobile phases consisted of acetonitrile and water (95:5) with 0.1% formic acid (mobile phase A), and methyl tert-butyl ether/acetonitrile with water (85:10:5) (mobile phase B), with a flow rate of 1.2 mL/min. The following gradient was employed (min/%A): (0/100%), (10/75%), (23/15%), (25/15%), and (27/100%). Carotenoids were detected at 450 nm and identified based on retention times of the respective analytical standards.

### 2.3. Genome-Wide Association Study

A genome-wide association study (GWAS) was conducted using the TASSEL v.5.2.93 software in the R package. The mixed linear model (MLM) was estimated without a compression level, and variance components were estimated once using re-estimation after each marker. The SNPs were filtered to remove markers with more than 20% missing data and a minor allele frequency (MAF) less than 0.05, thus ensuring the reliability of the estimates. The model was employed in conjunction with a marker-derived kinship and principal components, which were used to control the relationship and population structure, respectively. To identify significant SNPs, the threshold *p*-value was determined according to the Bonferroni correction method. The threshold *p*-value was calculated as 0.01–0.05/n, where n is the number of SNPs used in the GWAS. Manhattan plots and quantile-quantile (QQ) plots were generated using CMplot (v.1.0.1) from the R package. The proportion of phenotypic variation explained by each marker was estimated based on the explained phenotype variance. Allele frequencies and major and minor gamete frequencies were calculated using TASSEL software (version 5.2.93) with the default settings.

### 2.4. Population Structure Analysis and Linkage Disequilibrium (LD) Estimation

Phylogenetic trees, relative kinship, identity-by-state genetic distances between pairs of accessions, and principal component analysis (PCA) were performed using TASSEL v.5.2.93 software [33] to determine the genetic population structure of the samples and to calculate linkage disequilibrium (LD), expressed as r^2^. Permutation tests were employed to determine the degree of statistical significance associated with LD between loci. A pairwise LD analysis was conducted between the significant markers identified in the GWAS model, based on marker score correlations (r^2^) on the full panel. The results were visualized as a heatmap using the heatmap function in R. The LD decay curve was fitted to the scatterplot by smoothing the spline regression line at the genome level [34].

### 2.5. Identification and Analysis of Candidate Genes

Candidate genes were explored using a combination of significant SNPs of GWAS *p*-values, local linkage disequilibrium (measured as r^2^), and gene annotation using the gff3 file of the MU-CU-16 genome v4.1, which is available on CuGenDBv2A (http://cucurbitgenomics.org/v2/). A standard interval of 140 kb (69.7 kb upstream and 69.7 kb downstream) was explored for each candidate locus, with adjustments made according to the degree of local linkage disequilibrium with the candidate SNP (r^2^ < 0.2).

To analyze gene expression in different tissues, including the yellow–orange flesh of the Lady Godiva (LG) and Sweet REBA (SR) cultivars and the white flesh of the MUCU-16 cultivar, we used the previous RNA-seq projects PRJNA339848 and PRJNA1042934 from NCBI.

To investigate whether GWAS-mapped transcription factor (TF) genes could be involved in the regulation of carotenoid metabolism, a co-expression analysis between these TFs and carotenoid-related genes was performed using the different RNA-seq projects of our group in different tissues and conditions and the ARACNE algorithm [35]. The transcription factors were obtained from PlantTFDB [36]. Minimum mutual information (MI), the value used to measure the dependence between two random variables, was obtained, and interactions with *p*-values < 1 × 10^−4^ were selected after 100 bootstrap iterations [37].

### 2.6. Statistical Analyses

The data summary and descriptive statistics were calculated using Microsoft Excel 2019 (Microsoft Corporation, Redmond, WA, USA). All statistical analyses were performed using R software (version 4.4.2) with the R packages “ggplot2”(version 3.5.1) “psych” (version 2.4.6.26) and “ggbiplot” (version 0.6.2). The assessment of correlations among traits was conducted through Pearson’s correlation analysis. The statistical significance of the observed differences between genotypes and traits was determined by conducting a Wilcoxon signed-rank test. A *p*-value of ≤0.05 was considered to indicate statistical significance. Principal component analysis (PCA) was employed to illustrate the statistical correlation between genotypes in significant single-nucleotide polymorphisms (SNPs) and the lutein, xanthophyll, α-carotene, and β-carotene of each accession using R software (version 4.4.2).

## 3. Results

### 3.1. Phenotypic Variation, Frequency Distribution, and Correlation Analysis of Carotenoid Content

A diversity panel comprising 259 accessions of *C. pepo* (Table S1), representing the genetic variability of the crop, was cultivated in 2022 under standard greenhouse conditions. Carotenoid content was quantified in a minimum of three biological replicates per accession. Those accessions whose carotenoid content was found to be variable between fruits of the same accession and those from which an insufficient number of fruits were obtained were excluded from the study. Ultimately, a panel of 120 accessions with robust phenotyping data was selected for the genome-wide association study (Table S1).

**Figure 1:**
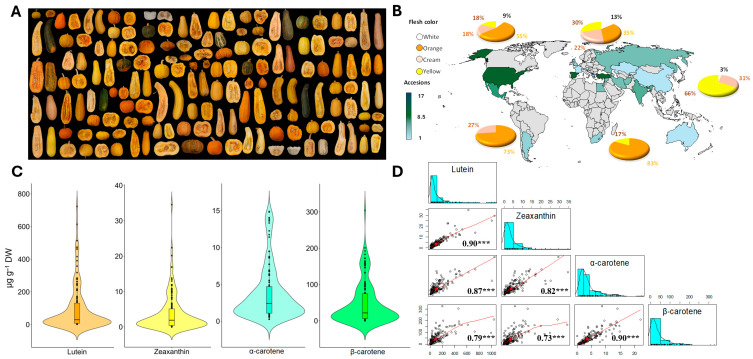
Phenotypic variation in the GWAS diversity panel consisting of 120 accessions of *C. pepo*. (**A**) Examples of fruit cross-sections from the panel showing the diversity in the color of the flesh of the fruit. (**B**) Worldwide distribution of the panel, showing the percentage of cultivars within each category of flesh color. (**C**) Frequency distributions of lutein, zeaxanthin, α-carotene, and β-carotene contents in the mature flesh. (**D**) Correlation matrices between the carotenoid contents. The middle diagonal contains histograms of the concentrations of each trait (n = 120). Scatter plots below the diagonal line show Pearson’s correlations between each pair of carotenoid contents. *** *p* < 0.001. Units = μg g^−1^ DW.

The squash diversity panel included all major morphological morphotypes and geographic centers of diversity worldwide (Figure 1A,B). The largest number of accessions was from Europe, with 60 accessions, while the smallest number was from Africa, with six accessions. The panel included samples of all flesh colors, including orange, cream, yellow, and white. The most common flesh color in the panel was orange, represented by 83%, 73%, and 55% of the accessions in Africa, Latin America, and the USA, respectively. This was followed by cream, yellow, and white flesh (Figure 1B). In Europe, 35% and 30% of the accessions exhibited orange and yellow flesh, respectively, while in Asia, 66% displayed yellow flesh. White flesh was the least common color in the panel (Figure 1B).

The concentrations of lutein, zeaxanthin, β-carotene, and α-carotene were quantified in the flesh of the fruit of the 259 squash accessions using HPLC (Table S1), allowing the determination of the range of carotenoid concentrations and their covariation with each other. For the 120 selected accessions, a wide range of variations were found for lutein, zeaxanthin, β-carotene, and α-carotene (Figure 1C, Table S1). Lutein was the most abundant carotenoid found in the squash fruits, with a range of 1.19–722.95 μg g^−1^ dry weight (DW) and a mean of 95.22 μg g^−1^ DW. β-carotene was the next most abundant carotenoid, with a range of 0.11–304.13 μg g^−1^ DW and a mean of 47.27 μg g^−1^ DW. The lowest concentration was observed for zeaxanthin (range, 0.00–34.56 μg g^−1^ DW; mean, 3.83 μg g^−1^ DW), followed by α-carotene (range, 0.27–14.88 μg g^−1^ DW; mean, 3.73 μg g^−1^ DW). There were strong positive correlations between the contents of lutein and zeaxanthin (r = 0.90, *p* < 0.001), α-carotene and β-carotene (r = 0.90, *p* < 0.001), lutein and α-carotene (r = 0.87, *p* < 0.001), zeaxanthin and α-carotene (r = 0.82, *p* < 0.001), lutein and β-carotene (r = 0.79, *p* < 0.001), and zeaxanthin and β-carotene (r = 0.73, *p* < 0.001) (Figure 1D).

### 3.2. Genetic Variation and Population Structure

**Figure 2:**
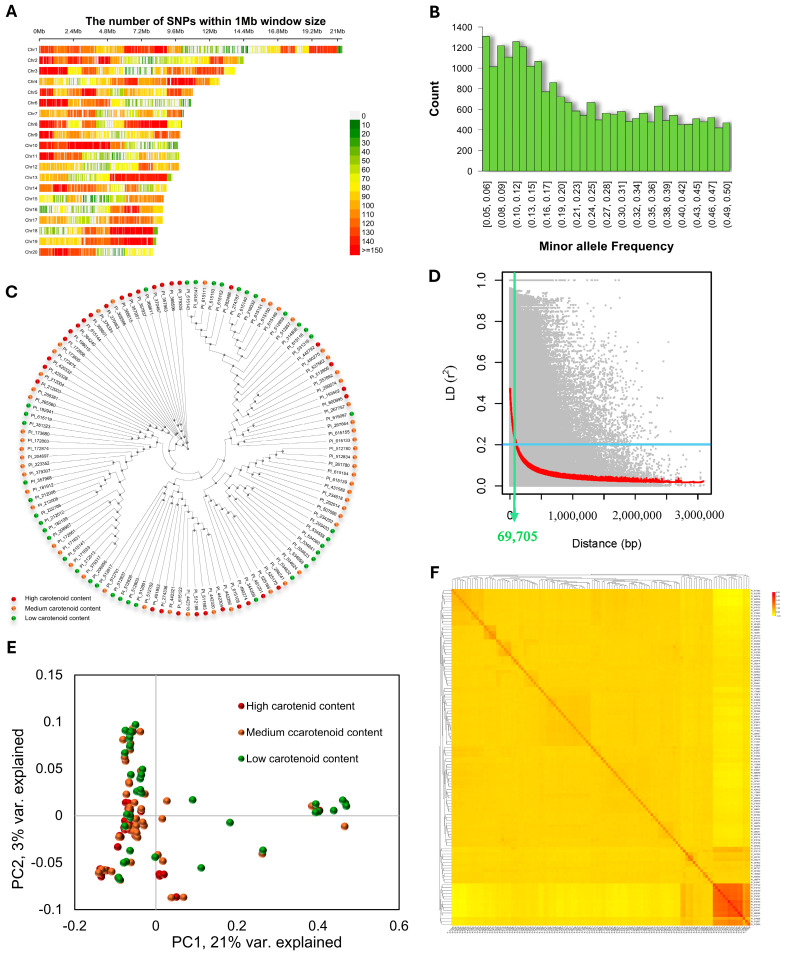
Overview of the genotypic dataset in the squash diversity panel. (**A**) SNP density plot of the 20 squash chromosomes representing the number of SNPs within a 1 Mb window size. The horizontal axis shows the chromosome length (Mb); the different colors represent SNP density. (**B**) Histogram of the minor allele frequency distribution of the squash panel. (**C**) Phylogenetic tree of the 120 squash accessions inferred from the total number of informative markers. The color of each accession denotes its phenotypic class according to carotenoid content. (**D**) Genome-wide linkage disequilibrium (LD) decay measured as r^2^ between intrachromosomal maker pairs versus the physical map distance between them (bp). The red smoothed curve (LOESS) and the average LD were fitted to the LD decay. (**E**) An assessment of the population structure of the squash panel based on principal components analysis (PCA) with 23,111 SNP markers. (**F**) Heatmap showing a pairwise kinship matrix.

The GBS data for the 120 selected accessions were obtained from CuGenDBv2A (http://cucurbitgenomics.org/v2/). After filtering out markers with a minor allele frequency of less than 5% and those with missing data of more than 20%, a total of 23,111 informative SNP markers were retained as the genetic dataset for this study. All SNPs were randomly distributed across the 20 chromosomes of the MUCU16 genome v4.1 with an average SNP density of one SNP every 9.19 Kb (Table S2; Figure 2A). The marker density per chromosome was variable. The highest marker density was observed on chromosome 19, with one SNP occurring every 7.89 Kb, while the lowest density was observed on chromosome 15, with one SNP occurring every 13.10 Kb (Table S2). The average minor allele frequency was 0.231, and 63% of the SNPs had an allele frequency greater than 0.1, indicating enrichment of common SNP alleles in the panel (Figure 2B).

Different approaches were used to infer the population structure and genetic relatedness between individuals in the squash germplasm panel that could result in false-positive and false-negative associations due to population stratification. The population structure did not seem to affect our study. The phylogenetic tree inferred from the total number of SNPs clearly showed that the 120 accessions were not clustered according to their carotenoid content, and, therefore, that the phenotypic classes are not genetically related (Figure 2C). To estimate the mapping resolution for our panel, we assessed the pairwise linkage disequilibrium (LD) between the 23,111 SNPs across the 120 squash accessions (Figure 2D). We used the mean r^2^ value as an estimate of LD decay using a 1 Mbp window, followed by fitting a non-linear regression curve of LD versus the genome’s physical distance. LD decay in this squash panel was, on average, 69.7 Kb based on r^2^ = 0.2 (Figure 2D). Given the genome size of ~260 Mb, a genome-wide LD decay of 69.7 Kb indicates that the minimum number of markers required to scan the entire squash genome would be approximately 3500, far fewer than the 23,111 SNPs used in this study.

Principal component analysis (PCA) was also performed on the whole-genome SNP dataset to visualize the extent and degree of population structure present within the panel. The first two principal components (PCs) together explained 23.1% of the total phenotypic variation, while the remaining principal components (PC3 to PC10) together explained approximately 15.64% of the total variation (Figure 2E). A remarkable degree of overlap was observed between accessions with different carotenoid contents, with no discernible clusters or subpopulations within the panel. The degree of relatedness between the accessions in the panel was estimated using the kinship matrix, shown as a heatmap in Figure 2F. A hierarchical tree was displayed among the accessions in the panel on the basis of their relatedness. Familial relationships were observed along the diagonal, with a few large blocks of closely related accessions in the panel. The off-diagonal portion of the relationship matrix indicated low relatedness among accessions in the diversity panel used in this study. Taken together, these results indicate that the population structure and genetic relatedness among accessions of the selected squash germplasm panel should not interfere with the whole-genome association study.

### 3.3. Genome-Wide Association Study of Carotenoid Content

**Figure 3:**
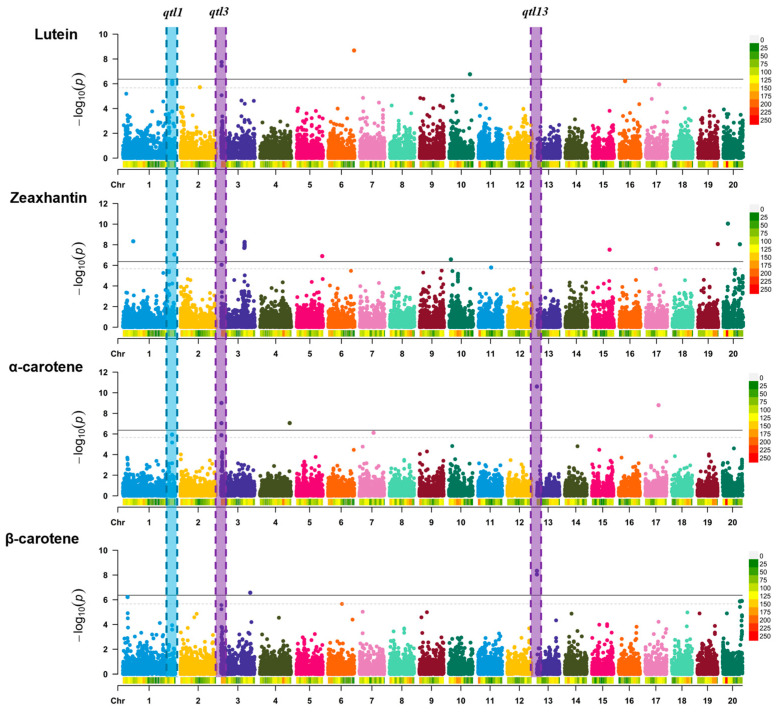
Manhattan plots showing the significant associations between SNPs in different genomic regions and the carotenoid content in the flesh of the fruit, including lutein, zeaxanthin, α-carotene, and β-carotene. The x-axis represents the chromosomal locations, and the y-axis shows the *p*-values of the association study on a logarithmic scale. Horizontal lines indicate the Bonferroni-corrected significance thresholds (dashed line, *p* < 0.05; solid line, *p* < 0.01) for the associations between SNPs and traits. The vertical-colored columns across the chromosomes correspond to six genomic loci associated with at least two carotenoid contents.

A genome-wide association study (GWAS), conducted using 23,111 high-quality single-nucleotide polymorphisms (SNPs), identified a total of 45 SNPs that were significantly associated with carotenoid content, with *p*-values lower than the Bonferroni thresholds (*p* < 0.05 or *p* < 0.01). The details of the identified SNPs are presented in Table S3 and illustrated in the Manhattan plots of Figure 3. Significant SNPs were distributed in seven chromosomal regions for lutein, six for α-carotene, five for β-carotene, and nine for zeaxanthin (Table S3). Given the high correlation between carotenoid levels, we narrowed down the identified SNPs by selecting genomic regions that were significantly associated with two or more carotenoid contents. Among the significant SNPs, we identified three loci associated with more than one carotenoid content (Table 1). They were located on chromosomes 1 (*qtl1*), 3 (*qtl3*), and 13 (*qtl13*) (Figure 3 and Table 1).

The phenotypic variance explained by each of the significant SNPs ranged from 18% to 37%, indicating potential genes controlling carotenoid content in the vicinity of the identified SNPs (Table 1). The significant SNPs in *qtl1* (S01_20043687, S01_20043688, and S01_20048481) were associated with lutein and α-carotene and explained 14–21% of the phenotypic variance (PVE) in the panel (Table 1). The significant SNPs in *qtl3* (S03_180136, S03_180626, S03_180668, S03_185870, and S03_185886) were associated with lutein, zeaxanthin, and α-carotene and explained 16–34% of the phenotypic variation, with α-carotene being the major contributor (Table 1). Two markers on chromosome 13 (S13_98826 and S13_101541) explained 37% of the phenotypic variation for α-carotene and 36% of the phenotypic variation for β-carotene (Table 1).

### 3.4. Estimation of Local LD and Identification of Candidate Genes

**Figure 4:**
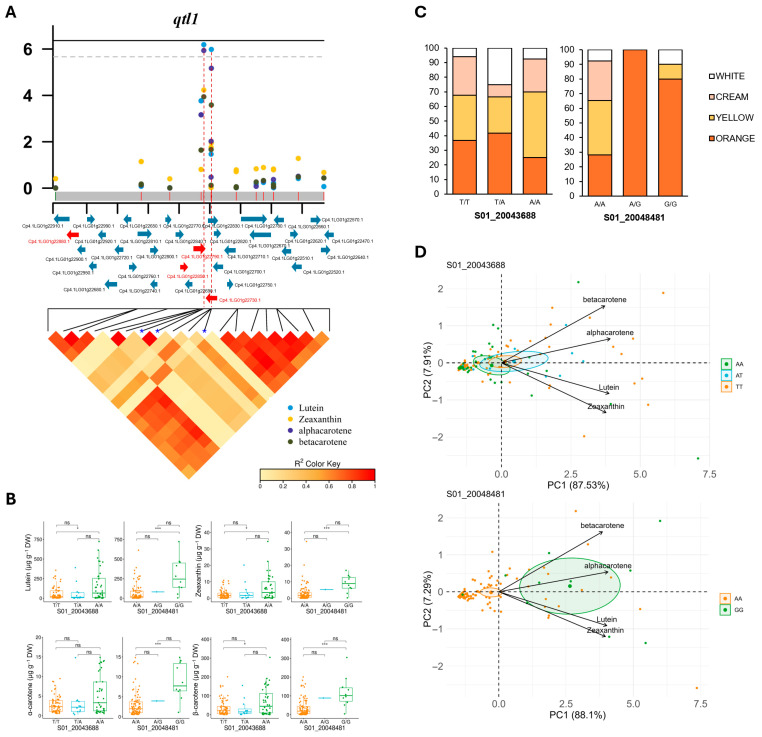
Regional association between the SNPs on chromosome 1 and carotenoid content. (**A**) Local Manhattan plots and linkage disequilibrium (LD) heatmap of the significantly associated genomic region on chromosome 1 (*qtl1*). The horizontal lines indicate the Bonferroni-corrected significance thresholds (dashed line, *p* < 0.05; solid line, *p* < 0.01). The region was defined as 69.5 Kb on either side of the most significantly associated SNP, with the average LD decay in the squash panel used for GWAS. Genes in the region are shown in blue, while candidate genes are shown in red. Red dashed lines indicate the genome positions of the most significant SNPs. The blue arrows indicate the genes in the genomic region, while the red arrows indicate candidate genes that may be associated with carotenoid accumulation. (**B**) Allelic effect boxplots of each significantly associated SNP with different carotenoid contents. Only those SNPs showing significant differences in carotenoid levels between genotypes of each SNP are shown. ns, *p* < 0.1; * *p* < 0.05; *** *p* < 0.001. For those SNPs that were very close to each other, only one allele is shown because the others had the same effect. (**C**) Bar plots showing the allelic effect of each significantly associated SNP on flesh color. (**D**) Principal component analysis (PCA) showing the variation in the 120 accessions of the GWAS diversity panel based on the genotype of significant *qtl1* SNPs and carotenoid content. Circles of distinct colors denote significant differences between the genotypes of one particular SNP (*p* ≤ 0.05).

Based on the average genome-wide LD decay (Figure 2D), a standard genomic interval of 140 Kb was used around the most significant SNP in each candidate locus to search for candidate genes associated with the contents of lutein, zeaxanthin, α-carotene, or β-carotene in the flesh of the fruit (69.7 kb upstream and 69.7 kb downstream of the most significant SNP). In the genomic interval of each candidate locus, the LD heatmap was used to display pairwise LD estimates between each pair of SNPs (Figure 4). Around the peak of each associated locus, the analysis revealed a decline in LD to intervals shorter than the genome-wide average of 69.7 Kb (Figure 2D), thus reducing the windows that should include the causative genes of the variation. In fact, significant SNPs in a given locus were as far apart as 5 Kb in *qtl1* and *qtl3* and 50 Kb in *qtl13* (Table S4). The candidate genes within these three loci are shown in Table 2.

Three significant markers were found in *qtl1* (Table 1 and Figure 4A). S01_20043687 and S01_20043688 were strongly linked, while S01_20048481 was only 5 Kb away from the other two. The local LD in the *qtl1* genomic interval of approximately 140 Kb was not very high (Figure 4A and Table 2). Therefore, although the interval includes 33 genes, the causal genes in this locus should be closer to the peak marked by the most significant SNPs. The three significant markers of *qtl1* were located in the linked genes Cp4.1LG01g22690 and Cp4.1LG01g22730, which encode a SPX domain-containing protein and phosphate transporter PHO1 homolog 3-like, respectively, both involved in phosphate homeostasis and signaling at the cellular and whole-plant levels and responsive to inorganic phosphate (Pi) starvation, but also in fruit carotenoid accumulation [38,39,40,41]. *qtl1* also contains the gene Cp4.1LG01g22880, which encodes protease Do-like 8 (DegP8/PDL8), a stroma-localized serine protease/chaperone that acts as a central player in chloroplast protein quality control (Table 2). The alternative alleles of the most significant SNPs in *qtl1* significantly increased the contents of lutein, zeaxanthin, α-carotene, and β-carotene (Figure 4B). Accordingly, most accessions that were heterozygous or homozygous for the alternative allele of SNP S01_20048481 (A/G or G/G) had orange flesh (Figure 4C). Furthermore, principal component analysis (PCA) based on the carotenoid content and SNP S01_20048481 clearly separated the 120 accessions of the diversity panel into two significant groups: One including accessions homozygous for the reference allele (AA) and fruits with a reduced carotenoid content, and another including accessions homozygous for the alternative allele (GG) and fruits with high carotenoid levels (Figure 4D).

**Figure 5:**
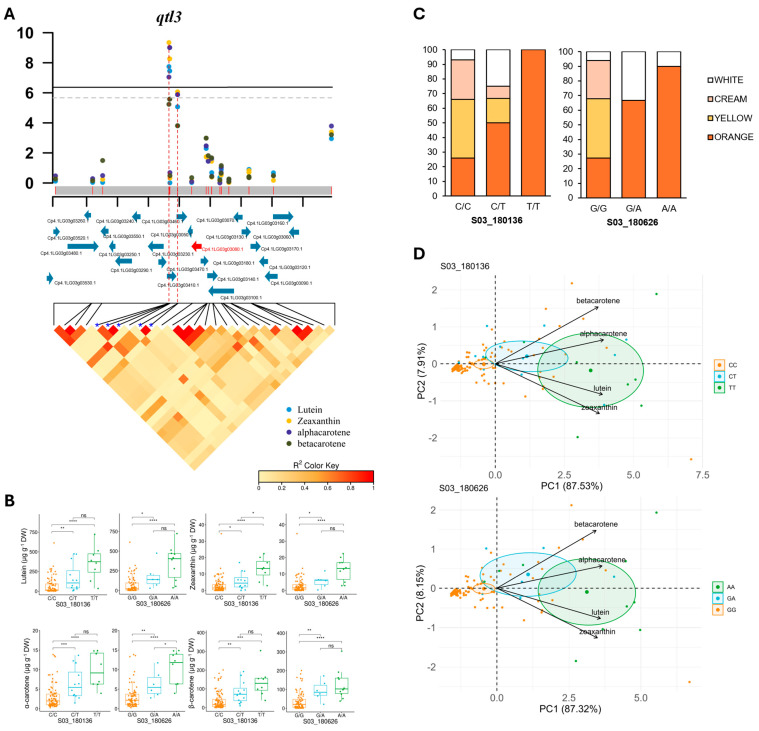
Regional association between the SNPs on chromosome 3 and carotenoid content. (**A**) Local Manhattan plot and linkage disequilibrium (LD) heatmap of the significantly associated genomic region on chromosome 3 (*qtl3*). Horizontal lines indicate the Bonferroni-corrected significance thresholds (dashed line, *p* < 0.05; solid line, *p* < 0.01). The region was defined as 69.5 Kb on either side of the top associated SNP, with the average LD decay in the squash panel used for GWAS. Genes in the region are shown in blue, while candidate genes are shown in red. Red dashed lines indicate the genome positions of the most significant SNPs. The blue arrows indicate the genes in the genomic region, while the red arrows indicate candidate genes that may be associated with carotenoid accumulation. (**B**) Allelic effect boxplots of each significantly associated SNP with different carotenoid contents. Only those SNPs showing significant differences in carotenoid levels between genotypes of each SNP are shown. ns, *p* < 0.1; * *p* < 0.05; ** *p* < 0.01; *** *p* < 0.001; **** *p* < 0.0001. For those SNPs that were very close to each other, only the allele of one is shown because the others showed the same effect. (**C**) Bar plots showing the allelic effect of each significantly associated SNP on flesh color. (**D**) Principal component analysis (PCA) showing the variation in the 120 accessions of the GWAS diversity panel based on the genotype of significant *qtl1* SNPs and carotenoid content. Circles of distinct colors denote significant differences between the genotypes of one particular SNP (*p* ≤ 0.05).

Five linked SNPs were significantly associated with carotenoid content in a region of approximately 5 Kb in *qtl3* (Table 1 and Figure 5A). The SNPs were located in an LD block containing the gene Cp4.1LG03g03080, which encodes TIC56, a component of the 1-megadalton translocon in the inner membrane of chloroplasts (Figure 5A and Table 2). The TIC protein complex interacts with ORANGE (OR) and other chaperones such as HEAT SHOCK PROTEINS 70, 90 and 93 (HSP70/90/93), involved in the quality control of chloroplast proteins, including enzymes involved in carotenoid biosynthesis. We also found that the alternative alleles of two SNPs on *qtl3* significantly increased the contents of lutein, zeaxanthin, and α- and β-carotene in the flesh (Figure 5B), confirming the role of this locus and the candidate gene TIC56 in the regulation of carotenoid content in pumpkin flesh. Moreover, orange flesh accessions were found to be mostly heterozygous or homozygous for the alternative alleles of these two SNPs (Figure 5C). PCA offered a visual representation of the diversity panel studied based on fruit carotenoid content (Figure 5D). The accessions homozygous for the reference alleles were clustered in the left part of the plot, while the accessions homozygous for the alternative allele were clustered in the right half of the plot due to their high carotenoid contents. Heterozygous accessions with an intermediate level of carotenoids were clustered in the central part of the PCA plot (Figure 5D). These data indicate that the alternative allele of *qtl3* SNPs confers a higher carotenoid content and an orange color on the pumpkin flesh.

**Figure 6:**
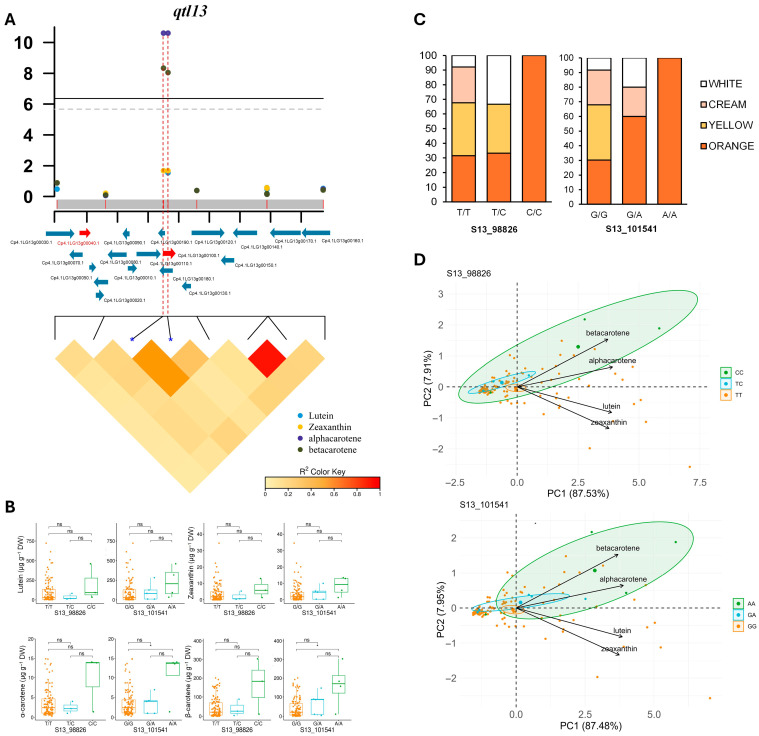
Regional association between the SNPs on chromosome 13 and carotenoid content. (**A**) Local Manhattan plot and linkage disequilibrium (LD) heatmap of the significantly associated genomic region on chromosome 13 (*qtl13*). Horizontal lines indicate the Bonferroni-corrected significance thresholds (dashed line, *p* < 0.05; solid line, *p* < 0.01). The region was defined as 69.5 Kb on either side of the top associated SNP, with the average LD decay in the squash panel used for GWAS. Genes in the region are shown in blue, and candidate genes are shown in red. The linkage disequilibrium (LD) heatmap is shown for each locus. Red dashed lines indicate the genome positions of the most significant SNPs. The blue arrows indicate the genes in the genomic region, while the red arrows indicate candidate genes that may be associated with carotenoid accumulation. (**B**) Allelic effect boxplots of each significantly associated SNP with different carotenoid contents. Only those SNPs showing significant differences in carotenoid levels between the genotypes of each SNP are shown. ns, *p* < 0.1; * *p* < 0.05. For those SNPs that were very close to each other, only the allele of one is shown because the others showed the same effect. (**C**) Bar plots showing the allelic effect of each significantly associated SNP on flesh color. (**D**) Principal component analysis (PCA) showing the variation in the 120 accessions of the GWAS diversity panel based on the genotype of significant *qtl1* SNPs and carotenoid content. Circles of distinct colors denote significant differences between the genotypes of one particular SNP (*p* ≤ 0.05).

In *qtl13,* we detected two SNPs that were significantly associated with both α- and β-carotene levels (Table S4). The two SNPs (S13_98826 and S13_101541) were located approximately 45 Kb apart from the Cp4.1LG13g00040 gene but within the same LD block (Figure 6A and Table 2). This gene encodes chloroplast HSP70, a chaperone known to regulate the accumulation and stability of enzymes involved in carotenoid biosynthesis [42]. In the panel of accessions used, SNPs in this gene were found to be associated with the flesh color of the fruit and the carotenoid content. The accessions homozygous for the reference alleles were fruits with white or cream flesh and low α- and β-carotene contents, including the reference genome accession MUCU16, whereas the few accessions homozygous for the alternative allele all had orange flesh with high α- and β-carotene contents (Figure 6C). Therefore, HSP70 is a strong candidate gene for *qtl13*. Sixteen other genes were annotated in the approximately 140 Kb genomic interval of *qtl13*. Significant SNPs were located in the genes Cp4.1LG13g00180 and Cp4.1LG13g00100, which encode for an ATG4-like cysteine protease and a pentatricopeptide repeat-containing protein (Table 2 and Table S4), the latter being part of a large gene family, with some of its members regulating carotenoid accumulation in fruit. PCA also separated accessions of the diversity panel based on the genotype of *qtl13* significant SNPs, although accessions homozygous for the alternative alleles of these SNPs showed very high variation in flesh carotenoid content (Figure 6D).

### 3.5. Expression Profiles of the Candidate Genes

To validate the possible function of the candidate genes identified by the GWAS, we took advantage of several previous RNA-seq projects to analyze the gene expression in different tissues, including the yellow–orange flesh of the Lady Godiva (LG) and Sweet REBA (SR) cultivars and the white flesh of the MUCU-16 cultivar. The orange fruits of the LG and SR cultivars accumulate lutein and β-carotene during ripening [43], whereas the fruits of the MUCU-16 cultivar accumulate very low levels of carotenoids during development (unpublished work). The results are shown in Figure S1. All genes were found to be expressed in the fruit, confirming their putative function in the accumulation of carotenoids in the fruit. It is also noteworthy that the expression profile of the Cp4.1LG01g22850 (*CpMYB106*) gene is induced during ripening of LG and SR fruits, reinforcing their possible involvement in carotenoid accumulation.

To investigate whether GWAS-mapped transcription factor (TF) genes could be involved in the regulation of carotenoid metabolism, a co-expression analysis between these TFs and carotenoid-related genes was performed using the different RNA-seq projects of our group in different tissues and conditions and the ARACNE algorithm [37]. The only TF that was found to be co-expressed with carotenoid biosynthesis genes (Cp4.1LG04g01620 and Cp4.1LG08g06310) was Cp4.1LG01g22850 (*CpMYB106*) in *qtl1* (Table S4). This gene was therefore included in the list of candidate genes regulating carotenoid accumulation in the fruit (Table 2).

## 4. Discussion

Despite the increasing nutraceutical importance of carotenoids, the basic genetic mechanisms controlling carotenoid metabolism in *C. pepo* fruit remain unknown. The formation of color associated with the ripening of fleshy fruits in numerous species is predominantly dependent on the differentiation and activity of chromoplasts, which are specialized organelles generated from plastids and rich in carotenoid pigments [44]. In this study, we used 120 diverse accessions collected from around the world to assess the phenotypic variation in carotenoid content and to investigate the underlying genetic variations that regulate the accumulation of carotenoids in the flesh of the fruit. Lutein was found to be the carotenoid accumulated the most in the pulp of the different accessions analyzed, followed by β-carotene. Similar carotenoid contents to those observed in this study have been reported in yellow-fleshed fruit [45] and in a collection of 100 selfed heirloom accessions and highly inbred lines of *C. maxima* [4,46].

The phenotypic data for carotenoid content were combined with genotyping by sequencing (GBS) data in the cucurbit genomic database CuGenDBv2 (http://cucurbitgenomics.org/v2/) for the GWAS, a genomic approach used to map important agronomic traits in different crop species [47,48,49]. This success was possible because of the enormous density of markers obtained by massive sequencing. On this occasion, the genotyping data consisted of 23,111 high-quality SNPs covering the 20 squash chromosomes with an average density of one SNP every 69.7 Kb. Given the size of the genome (~260 Mb) and the genome-wide LD decay estimated in this study (69.7 Kb), this marker density is theoretically sufficient for GWAS to identify variants associated with carotenoid content in this crop. Using the same genotyping data and a similar number of accessions, we recently used a GWAS to identify a QTL on chromosome 17 controlling postharvest chilling tolerance [50].

To validate the GWAS mapping, we estimated local LD in genomic regions surrounding significant SNPs associated with different carotenoid levels (Figure 4, Figure 5 and Figure 6). Allelic variation in many of these SNPs discriminates between accessions with high and low levels of the different carotenoids studied (Figure 4), which is useful for marker-assisted selection in breeding programs. We identified three loci on chromosomes 1, 3, and 13 associated with the accumulation of more than one carotenoid in the flesh, and the resolution of the GWAS mapping allowed the identification of several candidate genes controlling carotenoid accumulation in pumpkin flesh. We confirmed that all identified candidate genes were expressed in the fruit (Figure S1). In addition, the upregulation of Cp4.1LG01g22850 (*CpMYB106*) upon ripening of yellow/orange-fleshed fruits supports its possible role in the accumulation of carotenoids in pumpkin flesh.

The accumulation of carotenoids in plant organs depends on the translocation of carotenoid nucleus-encoded biosynthetic enzymes into the chloroplast, the maintenance of their proper folding and activity, and the rate of their degradation by chloroplast CLP proteases. Three of the most important candidate genes identified in *qtl1*, *qtl3,* and *qtl13* are related to chloroplast proteostasis: TIC56, HSP70, and DegP8/PDL8. TIC56 is a component of the translocon in the inner chloroplast membrane (TIC), which, together with the translocon in the outer chloroplast membrane (TOC), allows the transfer of proteins from the cytosol to the chloroplast stroma. The inner membrane channel interacts with members of the Hsp70 and Hsp90 stromal chaperone families, which act as ATP-dependent translocation motors for protein import and facilitate protein quality control and folding [51]. Therefore, it is likely that TIC56, HSP70, and DPL8 are capable of promoting the entry, folding, and degradation of enzymes involved in carotenoid biosynthesis. The chloroplast chaperones HSP70 and ORANGE (OR) not only contribute to protein folding and targeting but also deliver damaged proteins, including key carotenoid biosynthetic enzymes such as DSX and PSY, to the Clp and Deg proteases for deployment and degradation [42,52,53]. This would explain the increased PSY protein levels and carotenoid contents in Arabidopsis mutants for HSP70 and protease CLP [54], as well as the increased accumulation of lycopene and other carotenoids in transgenic fruits with silenced HSP70.2 isoforms in tomatoes [42]. Therefore, the chloroplast chaperone HSP70 in *qtl13* is likely a regulator of carotenoid accumulation in pumpkin flesh, as in Arabidopsis and tomatoes. The fact that the alternative alleles of the SNPs in *CpHSP70* confer yellow/orange-fleshed fruits with increased α- and β-carotene contents compared to the reference alleles in white-fleshed fruit accessions also supports this conclusion.

Recently, overexpression of the chromoplast inorganic phosphate (Pi) exporter PHT4;2 was shown to be required for flesh carotenoid accumulation and color development in watermelons [55] and citruses [56]. Several molecular mechanisms have been proposed to explain how efflux of Pi from the chromoplast to the cytoplasm can promote carotenoid accumulation, including increased ATP hydrolysis, enhanced chromoplast development, or enhanced expression of key genes in carotenoid biosynthesis [57]. Although the relationship between phosphate transport at the cell and whole-plant levels and carotenoid accumulation is uncertain, the GWAS mapping approach used in this work also highlighted the possible function of SPX domain-containing proteins and the PHO1-like phosphate transporter in *qtl1* for the differential accumulation of carotenoids in *C. pepo* fruit. Further research should be conducted to clarify the role of these enzymes in the flesh of fruits of this and other horticultural crops.

Pentatricopeptide repeat (PPR) proteins constitute a large family of RNA-binding proteins that play crucial roles in posttranscriptional modifications (editing, processing, stabilization, degradation, etc.) of mitochondria and chloroplast mRNA transcripts. They have been reported to be involved in organelle biogenesis and in the regulation of plant growth and development [58], but some chloroplast-localized PPR proteins have been found to be responsible for the flesh color variation and carotenoid accumulation in melons [59] and watermelons [60,61,62,63]. Therefore, it is likely that the PPR gene Cp4.1LG13g00100, detected in *qtl13*, also regulates the accumulation of carotenoids in pumpkin flesh. The increased contents of α-carotene and β-carotene we found in the fruits of homozygous accessions for the alternative allele of SNP S13_101541, located in Cp4.1LG13g00100, also support this conclusion.

## 5. Conclusions

There are currently no reliable studies on selecting plant materials and molecular markers for breeding new squash and pumpkin varieties with high antioxidant carotenoid contents. This study’s high-throughput phenotypic analysis of 257 *C. pepo* accessions identified carotenoid-rich plant materials that can be used to increase the lutein, zeaxanthin, α-carotene, and β-carotene levels in the flesh of mature fruit in new varieties. Additionally, a genome-wide association study (GWAS) using 23,111 high-quality SNPs and a diversity panel of 120 accessions identified three key loci on chromosomes 1, 3, and 13 associated with fruit carotenoid content in *C. pepo*. Several SNP markers were found to have pleiotropic effects on flesh color and the contents of multiple carotenoids, which could be useful in marker-assisted breeding. The identified candidate genes play essential roles in chloroplast proteostasis and carotenoid biosynthesis but also reveal novel regulatory mechanisms in carotenoid metabolism. These findings provide valuable insights into the genetic basis of fruit color and carotenoid accumulation in squash and lay the groundwork for future breeding programs aiming to improve the antioxidant capacity of *C. pepo* fruit.

## Figures and Tables

**Table 1:** Association statistics of the SNPs significantly associated with at least two carotenoid contents in each genomic locus (qtl).

Locus	SNP	Alleles	Lutein		Zeaxanthin		α-Carotene		β-Carotene	
			*p*-Value	PVE	*p*-Value	PVE	*p*-Value	PVE	*p*-Value	PVE
***qtl1***	S01_20043687	C/T	6.50 × 10^−7^	0.18	5.86 × 10^−5^	0.14	1.16 × 10^−6^	0.20	1.15 × 10^−4^	0.18
	S01_20043688	T/A	6.50 × 10^−7^	0.18	5.86 × 10^−5^	0.14	1.16 × 10^−6^	0.20	1.15 × 10^−4^	0.18
	S01_20048481	A/G	1.04 × 10^−6^	0.21	1.11 × 10^−2^	0.09	6.75 × 10^−6^	0.21	2.59 × 10^−4^	0.20
***qtl3***	S03_180136	C/T	1.80 × 10^−8^	0.24	4.53 × 10^−10^	0.29	8.96 × 10^−8^	0.23	5.83 × 10^−6^	0.23
	S03_180626	G/A	3.51 × 10^−8^	0.28	5.49 × 10^−9^	0.25	9.81 × 10^−10^	0.34	2.65 × 10^−6^	0.29
	S03_180668	C/T	3.51 × 10^−8^	0.28	5.49 × 10^−9^	0.31	9.81 × 10^−10^	0.34	2.65 × 10^−6^	0.29
	S03_185870	C/T	8.56 × 10^−6^	0.16	8.71 × 10^−7^	0.20	1.32 × 10^−6^	0.20	1.56 × 10^−4^	0.18
	S03_185886	T/C	8.56 × 10^−6^	0.16	8.71 × 10^−7^	0.20	1.32 × 10^−6^	0.20	1.56 × 10^−4^	0.18
***qtl13***	S13_98826	T/C	2.85 × 10^−2^	0.05	2.10 × 10^−2^	0.06	2.48 × 10^−11^	0.37	4.59 × 10^−9^	0.36
	S13_101541	G/A	2.85 × 10^−2^	0.05	2.10 × 10^−2^	0.06	2.48 × 10^−11^	0.37	8.91 × 10^−9^	0.36

Only SNPs that were significantly associated with the trait according to Bonferroni correction (green) are shown. PVE, phenotypic variance explained.

**Table 2:** Candidate genes linked to SNPs significantly associated with lutein, α-carotene, β-carotene, and zeaxanthin in the GWAS.

QTL	Gene	Description	Trait
***qtl1***	Cp4.1LG01g22880	Protease Do-like 8, chloroplast (DegP8/PDL8)	Lutein
	Cp4.1LG01g22850	Transcription factor MYB106-like	Zeaxanthin
	Cp4.1LG01g22790	SPX domain-containing protein	β-carotene
	Cp4.1LG01g22730	Phosphate transporter PHO1 homolog 9-like	α-carotene
***qtl3***	Cp4.1LG03g03080	Protein TIC 56, chloroplast	Lutein
			Zeaxanthin
			β-carotene
			α-carotene
***qtl13***	Cp4.1LG13g00040	Heat shock protein 70, chloroplast	β-carotene
	Cp4.1LG13g00100	Pentatricopeptide repeat (PPR) proteins	α-carotene

## Data Availability

All relevant data can be found within the manuscript and its Supporting Materials. The GBS data of *C. pepo* used in this study is available at the CuGenDBv2 database (http://cucurbitgenomics.org/v2/). The transcriptomic data were deposited in the NCBI-SRA database (https://www.ncbi.nlm.nih.gov/sra/). Project number: PRJNA1019120.

## Supplementary Materials

The following supporting information can be downloaded at https://www.mdpi.com/article/10.3390/antiox14091090/s1, Figure S1: Expression profiles of candidate genes controlling the carotenoid content in the flesh of the fruit. Expression was determined in the yellow/orange flesh of Lady Godiva (LG) and Sweet Reba (SR), and in the white flesh of MUCU16, obtained from RNA-seq projects PRJNA339848 and PRJNA1042934 from NCBI. Fruits 5, 15, and 40 represent immature, intermediate, and mature fruits at 5, 15, and 40 days after pollination. In the MUCU16 cultivar, expression data from other plant organs, including leaves, roots, and seeds, are also presented; Table S1: Geographical distribution, fruit color, and carotenoid content of the panel of accessions used in the GWAS; Table S2: Summary of the density of SNPs across 20 chromosomes of *C. pepo* MU-CU-16 genome v4.1; Table S3: Association statistics of SNPs significantly associated with carotenoid content according to the GWAS; Table S4: Candidate genes linked to SNPs associated with carotenoid content.
