# Supplementary material for: A Genome-Wide Association Study Reveals QTLs and Candidate Genes Associated with the Carotenoid Content in the Flesh of Cucurbita pepo L. Fruit
Source: Antioxidants (Basel). 2025 Sep 5;14(9):1090. doi: 10.3390/antiox14091090 (PMC12466424; doi:10.3390/antiox14091090)
Supplement: Supplementary file 1 [file antioxidants-14-01090-s001.zip › Figure S1.pptx]

## Slide 1
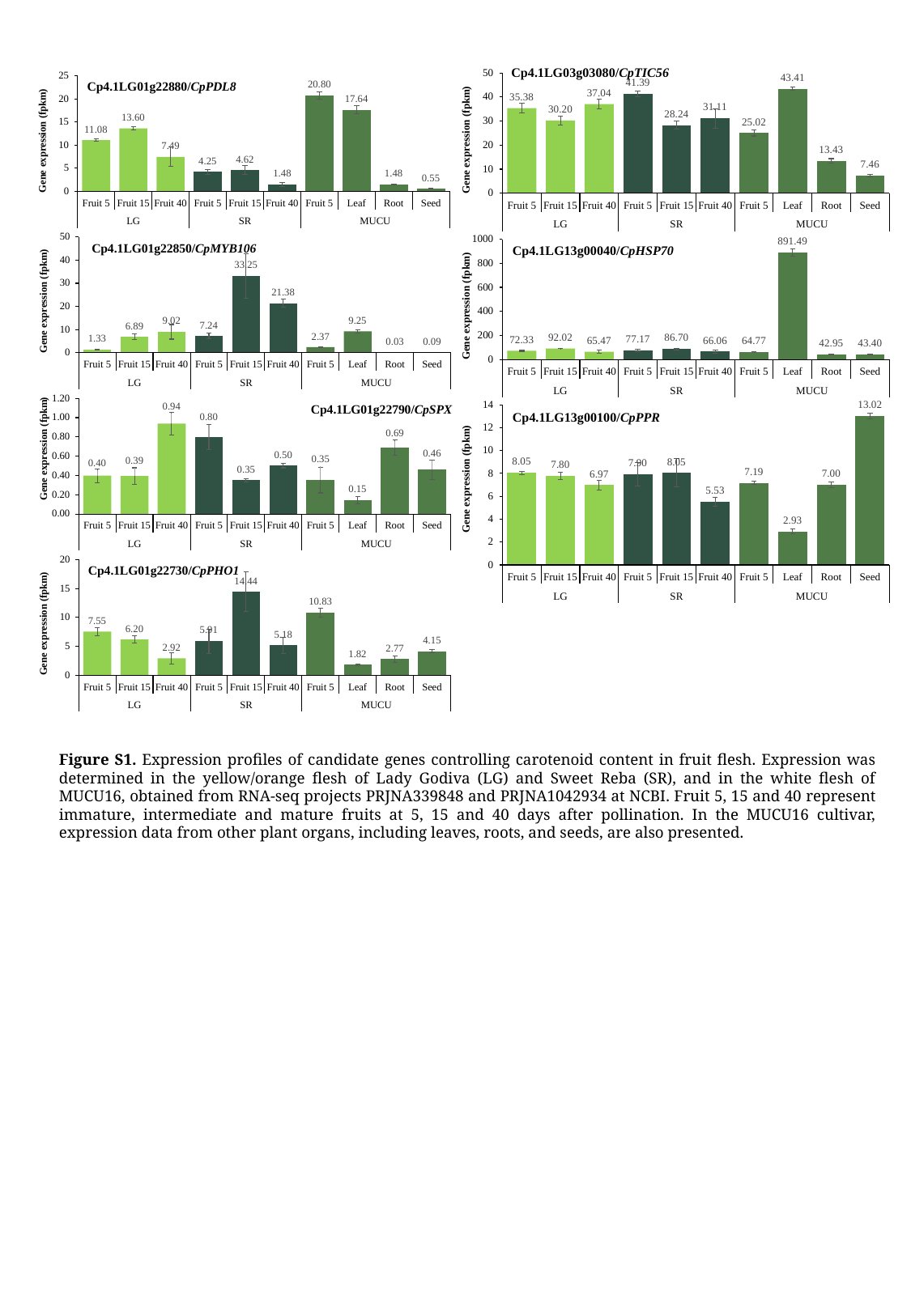

Figure S1. Expression profiles of candidate genes controlling carotenoid content in fruit flesh. Expression was determined in the yellow/orange flesh of Lady Godiva (LG) and Sweet Reba (SR), and in the white flesh of MUCU16, obtained from RNA-seq projects PRJNA339848 and PRJNA1042934 at NCBI. Fruit 5, 15 and 40 represent immature, intermediate and mature fruits at 5, 15 and 40 days after pollination. In the MUCU16 cultivar, expression data from other plant organs, including leaves, roots, and seeds, are also presented.
